# Supplementary material for: Unveiling the Bioactive Potential of the Invasive Jellyfish Phyllorhiza punctata Through Integrative Transcriptomic and Proteomic Analyses
Source: Biomolecules. 2025 Aug 4;15(8):1121. doi: 10.3390/biom15081121 (PMC12383608; doi:10.3390/biom15081121)
Supplement: Supplementary file 1 [file biomolecules-15-01121-s001.zip › Suplementary Material/FileS3/PEPFOLD4/XP_065064368_6851/clusters.html]

 PEP-FOLD clusters


Download archive of all models  here   

|  |  |  |
| --- | --- | --- |
| # | file | sOPEP |
| # cluster 1 - Size: 13 |  |  |
|  | XP\_065064368\_6851-model1 | -23.2288 |
|  | XP\_065064368\_6851-model1.1 | -23.1942 |
|  | XP\_065064368\_6851-model1.2 | -22.8147 |
|  | XP\_065064368\_6851-model1.3 | -21.7407 |
|  | XP\_065064368\_6851-model1.4 | -21.4111 |
|  | XP\_065064368\_6851-model1.5 | -20.6485 |
|  | XP\_065064368\_6851-model1.6 | -20.0809 |
|  | XP\_065064368\_6851-model1.7 | -19.9964 |
|  | XP\_065064368\_6851-model1.8 | -19.5372 |
|  | XP\_065064368\_6851-model1.9 | -19.3492 |
|  | XP\_065064368\_6851-model1.10 | -17.3712 |
|  | XP\_065064368\_6851-model1.11 | -16.2003 |
|  | XP\_065064368\_6851-model1.12 | -15.627 |
| # cluster 2 - Size: 6 |  |  |
|  | XP\_065064368\_6851-model2 | -23.1278 |
|  | XP\_065064368\_6851-model2.1 | -21.7245 |
|  | XP\_065064368\_6851-model2.2 | -20.9604 |
|  | XP\_065064368\_6851-model2.3 | -20.8604 |
|  | XP\_065064368\_6851-model2.4 | -20.6692 |
|  | XP\_065064368\_6851-model2.5 | -20.3501 |
| # cluster 3 - Size: 4 |  |  |
|  | XP\_065064368\_6851-model3 | -22.3108 |
|  | XP\_065064368\_6851-model3.1 | -21.7567 |
|  | XP\_065064368\_6851-model3.2 | -20.463 |
|  | XP\_065064368\_6851-model3.3 | -19.7153 |
| # cluster 4 - Size: 7 |  |  |
|  | XP\_065064368\_6851-model4 | -21.6012 |
|  | XP\_065064368\_6851-model4.1 | -21.3855 |
|  | XP\_065064368\_6851-model4.2 | -20.9223 |
|  | XP\_065064368\_6851-model4.3 | -20.7393 |
|  | XP\_065064368\_6851-model4.4 | -19.8311 |
|  | XP\_065064368\_6851-model4.5 | -18.4163 |
|  | XP\_065064368\_6851-model4.6 | -15.1082 |
| # cluster 5 - Size: 2 |  |  |
|  | XP\_065064368\_6851-model5 | -20.1885 |
|  | XP\_065064368\_6851-model5.1 | -19.3753 |
| # cluster 6 - Size: 3 |  |  |
|  | XP\_065064368\_6851-model6 | -19.3022 |
|  | XP\_065064368\_6851-model6.1 | -15.746 |
|  | XP\_065064368\_6851-model6.2 | -11.4843 |
| # cluster 7 - Size: 1 |  |  |
|  | XP\_065064368\_6851-model7 | -19.2254 |
| # cluster 8 - Size: 1 |  |  |
|  | XP\_065064368\_6851-model8 | -18.4937 |
| # cluster 9 - Size: 10 |  |  |
|  | XP\_065064368\_6851-model9 | -18.4893 |
|  | XP\_065064368\_6851-model9.1 | -17.7343 |
|  | XP\_065064368\_6851-model9.2 | -17.6763 |
|  | XP\_065064368\_6851-model9.3 | -17.6619 |
|  | XP\_065064368\_6851-model9.4 | -17.6619 |
|  | XP\_065064368\_6851-model9.5 | -17.4188 |
|  | XP\_065064368\_6851-model9.6 | -16.8421 |
|  | XP\_065064368\_6851-model9.7 | -15.6506 |
|  | XP\_065064368\_6851-model9.8 | -13.7862 |
|  | XP\_065064368\_6851-model9.9 | -12.6505 |
| # cluster 10 - Size: 14 |  |  |
|  | XP\_065064368\_6851-model10 | -18.4556 |
|  | XP\_065064368\_6851-model10.1 | -17.7468 |
|  | XP\_065064368\_6851-model10.2 | -17.6834 |
|  | XP\_065064368\_6851-model10.3 | -17.5814 |
|  | XP\_065064368\_6851-model10.4 | -17.3829 |
|  | XP\_065064368\_6851-model10.5 | -17.1743 |
|  | XP\_065064368\_6851-model10.6 | -16.9928 |
|  | XP\_065064368\_6851-model10.7 | -16.5491 |
|  | XP\_065064368\_6851-model10.8 | -16.3804 |
|  | XP\_065064368\_6851-model10.9 | -16.3089 |
|  | XP\_065064368\_6851-model10.10 | -16.1467 |
|  | XP\_065064368\_6851-model10.11 | -15.8682 |
|  | XP\_065064368\_6851-model10.12 | -13.9117 |
|  | XP\_065064368\_6851-model10.13 | -13.2495 |
| # cluster 11 - Size: 4 |  |  |
|  | XP\_065064368\_6851-model11 | -18.1765 |
|  | XP\_065064368\_6851-model11.1 | -17.4854 |
|  | XP\_065064368\_6851-model11.2 | -16.1663 |
|  | XP\_065064368\_6851-model11.3 | -13.4921 |
| # cluster 12 - Size: 3 |  |  |
|  | XP\_065064368\_6851-model12 | -18.1256 |
|  | XP\_065064368\_6851-model12.1 | -17.3514 |
|  | XP\_065064368\_6851-model12.2 | -16.038 |
| # cluster 13 - Size: 3 |  |  |
|  | XP\_065064368\_6851-model13 | -17.7185 |
|  | XP\_065064368\_6851-model13.1 | -16.5248 |
|  | XP\_065064368\_6851-model13.2 | -12.7104 |
| # cluster 14 - Size: 1 |  |  |
|  | XP\_065064368\_6851-model14 | -17.7081 |
| # cluster 15 - Size: 3 |  |  |
|  | XP\_065064368\_6851-model15 | -17.611 |
|  | XP\_065064368\_6851-model15.1 | -17.5372 |
|  | XP\_065064368\_6851-model15.2 | -17.225 |
| # cluster 16 - Size: 3 |  |  |
|  | XP\_065064368\_6851-model16 | -17.5504 |
|  | XP\_065064368\_6851-model16.1 | -15.7735 |
|  | XP\_065064368\_6851-model16.2 | -15.568 |
| # cluster 17 - Size: 1 |  |  |
|  | XP\_065064368\_6851-model17 | -16.9013 |
| # cluster 18 - Size: 2 |  |  |
|  | XP\_065064368\_6851-model18 | -16.6653 |
|  | XP\_065064368\_6851-model18.1 | -16.5195 |
| # cluster 19 - Size: 3 |  |  |
|  | XP\_065064368\_6851-model19 | -16.4089 |
|  | XP\_065064368\_6851-model19.1 | -16.2618 |
|  | XP\_065064368\_6851-model19.2 | -15.2974 |
| # cluster 20 - Size: 2 |  |  |
|  | XP\_065064368\_6851-model20 | -16.3808 |
|  | XP\_065064368\_6851-model20.1 | -15.8488 |
| # cluster 21 - Size: 2 |  |  |
|  | XP\_065064368\_6851-model21 | -16.3376 |
|  | XP\_065064368\_6851-model21.1 | -15.3463 |
| # cluster 22 - Size: 3 |  |  |
|  | XP\_065064368\_6851-model22 | -15.8827 |
|  | XP\_065064368\_6851-model22.1 | -13.5369 |
|  | XP\_065064368\_6851-model22.2 | -12.1046 |
| # cluster 23 - Size: 1 |  |  |
|  | XP\_065064368\_6851-model23 | -15.8491 |
| # cluster 24 - Size: 1 |  |  |
|  | XP\_065064368\_6851-model24 | -15.8158 |
| # cluster 25 - Size: 1 |  |  |
|  | XP\_065064368\_6851-model25 | -15.188 |
| # cluster 26 - Size: 1 |  |  |
|  | XP\_065064368\_6851-model26 | -14.5456 |
| # cluster 27 - Size: 1 |  |  |
|  | XP\_065064368\_6851-model27 | -13.4035 |
| # cluster 28 - Size: 1 |  |  |
|  | XP\_065064368\_6851-model28 | -13.1555 |
| # cluster 29 - Size: 1 |  |  |
|  | XP\_065064368\_6851-model29 | -12.5915 |
| # cluster 30 - Size: 1 |  |  |
|  | XP\_065064368\_6851-model30 | -12.439 |
| # cluster 31 - Size: 1 |  |  |
|  | XP\_065064368\_6851-model31 | -10.5819 |
|  |  |  |
